# Machine Learning-Guided Cycle Life Prediction for Electrochromic Devices Based on Deuterium and Water Mixing Solvent

**DOI:** 10.3390/mi15091073

**Published:** 2024-08-26

**Authors:** Yitong Wu, Sifan Kong, Qingxin Yao, Muyun Li, Huayi Lai, Duoyu Sun, Qingyue Cai, Zelin Qiu, Honglong Ning, Yong Zhang

**Affiliations:** 1 School of Electronics and Information Engineering, South China Normal University, Foshan 528225, China; yitongwu@m.scnu.edu.cn (Y.W.); 20213903020@m.scnu.edu.cn (Q.Y.); 2 School of Semiconductor Science and Technology, South China Normal University, Foshan 528225, China; 20225031032@m.scnu.edu.cn; 3 School of Software, South China Normal University, Foshan 528225, China; kongsifanscnu@foxmail.com (S.K.); 20222034018@m.scnu.edu.cn (D.S.); 4 State Key Laboratory of Luminescent Materials and Devices, School of Materials Science and Engineering, South China University of Technology, Guangzhou 510640, China; 13922705035@163.com (Q.C.); ninghl@scut.edu.cn (H.N.); 5 Aberdeen Institute of Data Science and Artificial Intelligence, South China Normal University, Foshan 528225, China; 20213802071@m.scnu.edu.cn

**Keywords:** electrochromism, machine learning, long short-term memory, cycle life

## Abstract

Electrochromic devices have demonstrated considerable potential in a range of applications, including smart windows and automotive rearview mirrors. However, traditional cycle life testing methods are time-consuming and require significant resources to process a substantial amount of generated data, which presents a significant challenge and remains an urgent issue to be addressed. To address this challenge, we proposed the use of Long Short-Term Memory (LSTM) networks to construct a prediction model of the cycle life of electrochromic devices and introduced an interpretable analysis method to further analyze the model’s predictive capabilities. The original dataset used for modeling was derived from preliminary experiments conducted under 1000 cycles of six devices prepared with varying mixing ratios of heavy water (D_2_O). Furthermore, validation experiments confirmed the feasibility of the D_2_O mixing strategy, with 83% of the devices exhibiting a high initial transmittance modulation amplitude (Δ*T* = 43.95%), a rapid response time (*t_c_* = 7 s and *t_b_* = 8 s), and excellent cyclic stability (Δ*T* = 44.92% after 1000 cycles). This study is the first to use machine learning techniques to predict the cycle life of electrochromic devices while proposing performance enhancement and experimental time savings for inorganic all-liquid electrochromic devices.

## 1. Introduction

Electrochromic devices, which change color when electricity is applied, are widely used in smart windows to save energy [1,2,3], aircraft portholes [4], automotive mirrors [5], and displays [6]. A crucial performance aspect of electrochromic devices is their cycle life. This indicates the maximum cycles a device can undergo before its performance declines. It is determined by the device’s sustained transmittance modulation amplitude (Δ*T*) over cycles [7,8,9], with a 30% reduction in Δ*T* typically indicating losses in electrochromic functionality [10]. For solid electrochromic devices, researchers are working to address the performance degradation relating to ion insertion and extraction by improving preparation methods (Δ*T* = 67.5% after 750 cycles) [11], material combinations (Δ*T* > 60% after 100 cycles) [12], and structural design (Δ*T* > 40% after 1300 cycles) [13] to extend their cycle life. In contrast, water-based liquid electrochromic devices have simple structures and anti-degradation advantages due to liquid mobility but also suffer from performance decline caused by hydrogen evolution reactions. Li et al. showed that heavy water (D_2_O) can extend cycle life via hydrogen bonding [14]. However, cycle life extension through the use of heavy water needs to be improved. In parallel, cycle life testing is challenging and data-heavy, requiring tools for more accessible research and insight [8,15,16].

As an indispensable tool in computer science, machine learning (ML) has been employed to analyze and interpret vast quantities of data [17]. Its application in materials science has garnered considerable interest in recent years [18,19,20,21,22]. Unlike the trial-and-error method, ML extracts correlations and material properties from preliminary experimental data to build models that guide research on material performance and longevity [23]. In numerous studies, ML techniques have been successfully employed to investigate the cycle life of various materials and devices, including batteries [24], quantum dots [25], titanium alloys [26], and chalcogenides [27]. Among these studies, the Long Short-Term Memory (LSTM) algorithm stands out for its proficiency in capturing long-term dependencies and processing sequential data, ideal for time-series forecasts and modeling degradation patterns. Zhang et al. developed an LSTM for lithium-ion battery RUL prediction, trained on degradation data from six batteries over 600 cycles. It outperformed traditional methods with errors confined to 10–20 cycles [28]. Similarly, John M. Howard et al. utilized an LSTM model to predict the photoluminescence of metal halide chalcogenides based on humidity and luminescence data, achieving an accuracy of over 89% within the next 12 h [29].

Currently, there is already some research applying ML in the field of simulation and prediction of the performance of electrochromic devices. Kong et al. developed high-performance electrochromic devices using a multilayer perceptron model to predict optimal concentrations from experimental data [30]. Faceira et al. utilized ML to predict the impact of sputtering parameters on the electrochromic properties of WO_3_ thin films, optimizing the fabrication process through a predictive model based on experimental data [31]. However, compared with its extensive use in analyzing and forecasting battery and chalcogenide cycle life, ML has not yet been applied to the cycle life analysis of electrochromic devices. Meanwhile, in existing studies on ML for predicting device cycle life, such as for batteries [32,33], most rely on publicly available datasets and lack experimental ML studies using exclusively self-collected data. It is also worth noting that Interpretable ML is increasingly emphasized at the intersection of materials science and computer science [34]. It provides researchers with deeper knowledge to guide experimental design and material optimization. In this context, the study of cycle life modeling of electrochromic devices incorporating interpretability is of particular importance.

In this study, we found a high cyclic stability all-liquid electrochromic device consisting of a hybrid functional layer synthesized from ammonium metatungstate and iron(II) chloride in a mixed solvent of deionized water (H_2_O) and D_2_O. This device achieved optimal transmission modulation amplitude (Δ*T* = 43.95%), swift response time (*t_c_* = 7 s and *t_b_* = 8 s), and long cycle life at an 83% D_2_O ratio (Δ*T* = 44.92% after 1000 cycles). The proposed D_2_O mixing strategy is able to assist the device in improving cycle life. In addition, based on the initial experimental data of the design, we constructed an LSTM model employed with a unique dataset, enabling the accurate prediction of the device’s transmittance over 500 cycles. We also enhance the interpretability of LSTM models by applying the locally interpretable model-agnostic interpretation (LIME) method. This study represents the first application of ML in the field of cycle life prediction for electrochromic devices and helps to accelerate the experimental development process.

## 2. Materials and Methods

**Figure 1:**
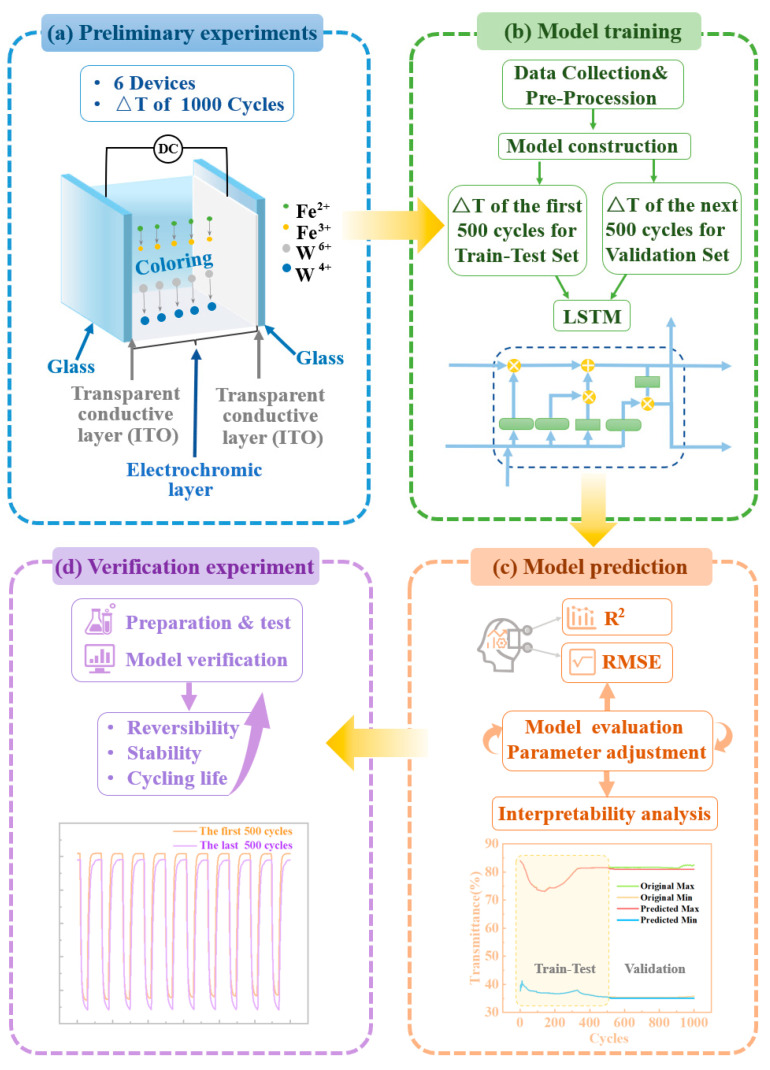
Flowchart of the development process for ML to predict the cycle life of electrochromic devices, including.

Figure 1 outlines our ML approach for predicting the cycle life of electrochromic devices. Six devices, each made with varying ratios of deionized to D_2_O, were tested for 1000 cycles at 45 A/m^2^ (Figure 1a). The normalized data were used to train an LSTM model, with the 1000-cycle time series split into a train–test set (first 500 cycles) and a validation set (last 500 cycles) (Figure 1b). The model refined parameters based on predictions to find the optimal solution (Figure 1c). Additional devices with new ratios were tested to validate the model (Figure 1d). Further details on the experiments and model construction will follow.

### 2.1. Solution Preparation

2 mL of solvent was added to the beaker, which was selected to be a mixture of heavy water (D_2_O, 99.9%, Jinan Mena Science and Technology Co., Ltd., Jinan, China) and H_2_O in different volume ratios. These ratios were 0%, 20%, 40%, 60%, 80%, and 100%, respectively. Ammonium metatungstate (AMT, (NH_4_)_6_H_2_W_12_O_40−x_H_2_O, 99.5%, Guangzhou Chemical Reagent Factory, Guangzhou, China) and ferrous chloride (FeCl_2_·4H_2_O, McLean Biochemicals Co. Ltd., Shanghai, China) were added to formulate 6 groups of different solutions. The concentration of ammonium metatungstate was maintained at 0.100 mol/L, while that of ferrous chloride was set at 0.150 mol/L. The mixed solutions were subjected to ultrasonic oscillation to facilitate the formation of precursor solutions. All chemical materials and reagents utilized throughout the solution preparation were employed in their original state without undergoing any further purification.

### 2.2. Device Fabrication

**Figure 2:**
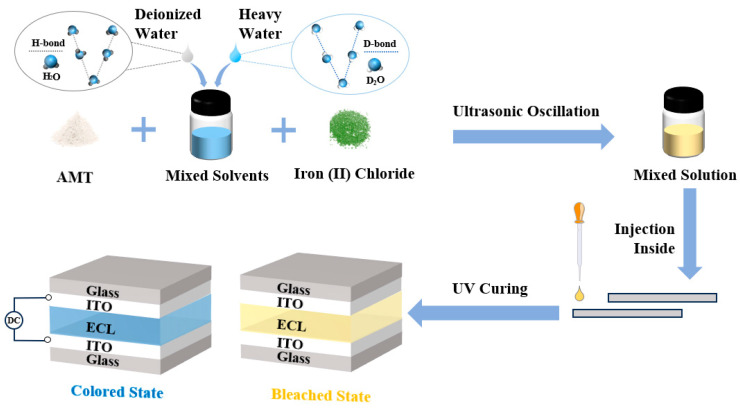
Flowchart of electrochromic device preparation.

Here, 4 × 4 cm^2^ indium tin oxide (ITO) glass substrates were cleaned with H_2_O and anhydrous ethanol for 15 min each and dried in an oven. Following this, two pieces of ITO glass were bonded with double-sided adhesive to form an effective area of 2 × 3 cm^2^. The solution was injected using the capillary method and finally fixed by applying UV-curable adhesive (see Figure 2). Following the above steps, the 6 prepared devices were uniformly named D-x, with x being the ratio of D_2_O mixing. The mixed functional layer had a thickness of 53.57 μm, as shown in Figure S1a.

### 2.3. Performance Characterization

The input current for the electrochromic experiments was provided by an electrochemical workstation (CH Instruments CHI660E, CH Instruments, Shanghai, China). A multi-current stepping (IStep) method was used to provide a constant current input, and 1000 cycles of current on/off tests were conducted at 0.027 A, corresponding to a current density of 45 A/m^2^, with a duration of 20 s for electrification and 40 s for power-off, making each cycle 60 s long. A micro-spectrometer (Morpho PG2000, Morpho, Shanghai, China) was used to record the change in transmittance over time. Since the main range for the optical transmittance spectra of the device in the initial state, colored state, and bleached state is 650–750 nm (see Figure S1b), we selected 700 nm as the reference wavelength.

### 2.4. Data Collection and Preprocessing

Following the D_2_O reference ratio, transmittance was measured over 1000 test cycles. Each cycle consisted of a 20 s charging phase followed by a 40 s rest phase, conducted at a current density of 45 A/m^2^. For each cycle, the maximum transmittance (*T_m_*) and minimum transmittance (*T_s_*) values were recorded and stored in the dataset.

To reduce the impact of data magnitude and outliers on model training, we applied min–max normalization to all the data. The relevant formulas are given in Equations (1) and (2) [35].

(1) $$T_{m}=\frac{T_{m}}{T_{mmax}}$$

(2) $$T_{s}=\frac{T_{s}}{T_{smax}}$$

In the above formula, *T_mmax_* is the maximum of *T_m_* in one 1000 times cycles test. Similarly, *T_smax_* is the maximum of *T_s_* in the cycle test.

### 2.5. Modeling Methods

**Figure 3:**
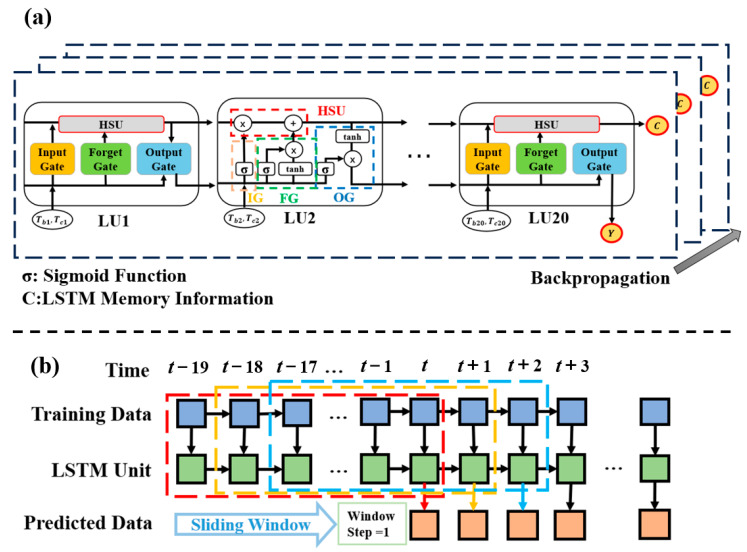
(**a**) Structure of the LSTM model used for cycle life prediction. (**b**) The m-1 structure of sliding windows in the LSTM neural network.

The LSTM neural network (shown in Figure 3a) was constructed to predict the results of future cycles of electrochromic testing based on the highest and lowest transmittance of the historical cycles of electrochromic testing.

LSTM is a specialized type of recurrent neural network (RNN) designed to learn long-range dependencies in sequential data effectively. Introduced by Hochreiter and Schmidhuber in 1997 [36], LSTM uses a unique gating mechanism consisting of input gates (IGs), forget gates (FGs), and output gates (OGs) to manage the flow of information through the network, and it uses a hidden state unit (HSU) in each LSTM unit (LU) to store all the sequential feature in the dataset. These characteristics have made LSTMs popular in various fields, including time series analysis [37], natural language processing [38], and computer vision [39]. LSTM is trained using gradient descent and backpropagation to minimize prediction error by optimizing model weights. Specifically, gradient descent updates weights in the direction of decreasing loss, while backpropagation computes the gradient by moving backward through the network, layer by layer, adjusting each weight to reduce the error.

The LSTM neural network we built contains 20 LU, each of which accepts 2 types of input data (the highest and lowest transmittance in a cycle test) and passes the data features to the subsequent LUs for storage in HSU, ultimately predicting the highest and lowest transmittance for the new cycle of testing at the 20th LU in conjunction with the existing training data.

In the realm of time-series forecasting, sliding window cross-validation emerges as a widely adopted strategy for assessing the performance of predictive models. This method is particularly suited for scenarios where sequentially leveraging historical data is crucial for enhancing predictive accuracy. In our study, we employed this technique to evaluate our predictive model for a given dataset comprising 1000 lines of data points, which were partitioned into a “train-test set” and a “validation set” on a 1:1 basis. Consequently, the initial 500 lines of data points were chosen to train and evaluate the model.

We implemented a “many-to-one” (m-1) sliding window structure (shown in Figure 3b) in the training and testing phases. This structure, as opposed to its “many-to-many” (m-m) counterpart, facilitates a more comprehensive utilization of past information by allowing each current input block to encompass overlapping data from the preceding block. Specifically, we set the window length to 20, with an output length of 1, and a sliding step size of 1. This configuration ensures that during each iteration, the model undergoes 480 successive window slides, and it can effectively capture temporal dependencies across the data sequence.

The adoption of the m-1 sliding window structure is motivated by its ability to capitalize on the overlapping information between consecutive input blocks. It is not only exposed to a broader context of historical data but can also better comprehend temporal patterns and dynamics within the series. In comparison to the m-m structure, where each output is independently influenced by a corresponding input time step, the m-1 structure enables the model to generate predictions that are inherently informed by the entirety of the current input block, thereby enhancing the overall predictive accuracy.

The model was trained in several iterations and tested on the training set, and R^2^ (Coefficient of Determination) and RMSE (Root Mean Square Error) were used as the evaluation metrics of the model’s fitting effectiveness. The combination of hyperparameters with the best performance is found by optimizing the maximum training iterations, batch size, loss function, and other model hyperparameters to maximize R^2^ and minimize RMSE. Grid search (GS) is a systematic hyperparameter tuning method that was used to find the best combination of hyperparameters for LSTM models. Based on measuring 6 sets of electrochromic devices with different concentrations of D_2_O added, we evaluated the fit of the system using a combination of R^2^ and RMSE and randomly fabricated one device that was different from the existing experiments for evaluation.

The implementation of the LSTM model is mainly based on Python 3.7.1, sci-kit-learn 1.0.2, and torch 1.13.1. The computer model used for experimental modeling is Legion Y9000X IAH7 with a 12th Gen Intel^®^ CoreTM i7-12700H CPU (Intel Corporation, Santa Clara, CA, USA), an NVIDIA GeForce RTX 3060 GPU, and 16 GB Samsung 4800 MHz DDR5 (Samsung, Seoul, Republic of Korea) memory.

## 3. Results and Discussion

### 3.1. Electrochromic Performance of the Devices

From the transmission spectrum shown in Figure S1b, it can be seen that the spectral curves of the initial and bleached states almost completely overlap. This indicates that the device has excellent reversibility and is able to quickly return to the original color after a power failure. In contrast, the memory effect refers to the phenomenon that the device maintains the color change state in the absence of a continuous electric field, which is usually manifested by the inability to fully return to the initial state during the fading process [40]. Since the spectral curves almost overlap, it indicates that the memory effect of the device is very small. This excellent reversibility and low memory effect ensures the long-term stability of the device.

Figure S2 displays the transmittance changes of the six devices during 1000 cycle life tests. Further, D-80 and D-100 were analyzed for Δ*T*, response time, and Δ*T* retention post-1000 cycles to understand the cycle life improvement mechanism. Δ*T* is the transmittance difference between the colored (*t_c_*) and bleached (*t_b_*) states, as detailed in Equation (3) [35].

(3) $$\Delta T=T_{b}-T_{c}$$

The response time is the duration for a device to transition between bleached and colored states, measured by a 90% optical transmittance change. Δ*T* retention is the ratio of post-cycle Δ*T* to the initial Δ*T* [41].

**Figure 4:**
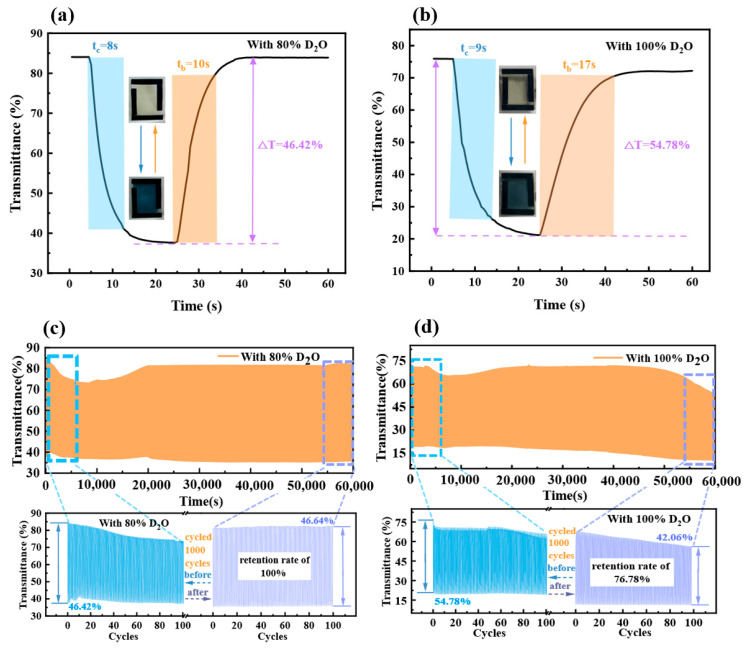
Transmittance–time characteristics and response time at 700 nm for (**a**) D-80 and (**b**) D-100. Transmittance change at 700 nm for (**c**) D-80 and (**d**) D-100 after 1000 cycles. Insets in (**a**,**b**) show the device in its initial and colored states.

Figure 4 illustrates that D-80 had an initial Δ*T* of 46.42%, *t_c_* of 8 s, and *t_b_* of 10 s, while D-100 had an initial Δ*T* of 54.78%, *t_c_* of 9 s, and *t_b_* of 17 s. After 1000 cycles, D-80’s Δ*T* slightly rose to 46.64%, while D-100’s dropped to 42.06% yet remained above 70%. Despite a lower initial Δ*T*, D-80 showed quicker response and better Δ*T* retention than their 100% counterparts.

This discrepancy may be attributed to the device’s reaction mechanism during the cycling process, as shown in Figure S3. The reactions involved are shown below. Equations (4) and (5) correspond to electrochromic reactions. Equations (6) and (7) correspond to the electrolysis of water and the combination of protons generated by H_2_O electrolysis with metatungstate to affect the discoloration of the device. Meanwhile, D_2_O electrolysis is shown in reaction 8, except that the degree of heavy water electrolysis is mild and almost negligible at low voltages [42,43,44].

(4) $${\left[H_{2}W_{12}O_{40}\right]}^{6-}+{\mathrm{xFe}}^{2+}+{\mathrm{ye}}^{-}={\left[H_{2}W_{12}O_{40}\right]}^{-6-x-y}+{\mathrm{xFe}}^{3+}\;(\mathrm{coloring})$$

(5) $${\left[H_{2}W_{12}O_{40}\right]}^{-6-x-y}+{\mathrm{xFe}}^{3+}-{\mathrm{ye}}^{-}={\left[H_{2}W_{12}O_{40}\right]}^{6-}+{\mathrm{xFe}}^{2+}\;(\mathrm{bleaching})\;$$

(6) $$nH_{2}O=2nH^{+}+\frac{n}{2}O_{2}↑$$

(7) $${\left[H_{2}W_{12}O_{40}\right]}^{6-}+2nH^{+}={\left[H_{2}W_{12}O_{40-n}\right]}^{6-}+nH_{2}O$$

(8) $$D_{2}O=D_{2}+\frac{1}{2}O_{2}↑$$

In the cycle life test, transmittance notably dropped, more so in the bleached state. During the coloring phase, the presence of electrolytic reactions increases the concentration of the bleaching solution. This leads to delayed particle deposition and bleaching recovery. The device’s Δ*T* initially fell but stabilized as hydrogen precipitation and D_2_O electrolysis reached equilibrium [43]. Performance initially declined due to memory effects and charge accumulation but stabilized with continued cycling. D-100 initially had a higher Δ*T*, but it decreased compared to D-80. The higher D_2_O content strengthened hydrogen bonds and stabilized D-O bonds, allowing higher voltage tolerance. However, high voltages can cause significant charge accumulation and reduce Δ*T* and may also lead to ammonium metatungstate crystallization, diminishing electrochromic properties. Identifying the optimal mix of D_2_O and H_2_O is crucial for enhancing device life and stability [14].

### 3.2. Modeling and Comparison

An aging model is essential to forecast the cycle life of electrochromic devices, updating performance data to capture long-term trends for accurate predictions. Four ML methods were tested to determine the best predictor of cyclic lifespan, which included LSTM, RNN, Bidirectional Recurrent Neural Network (Bi-RNN), and Gated Recurrent Unit (GRU).

**Figure 5:**
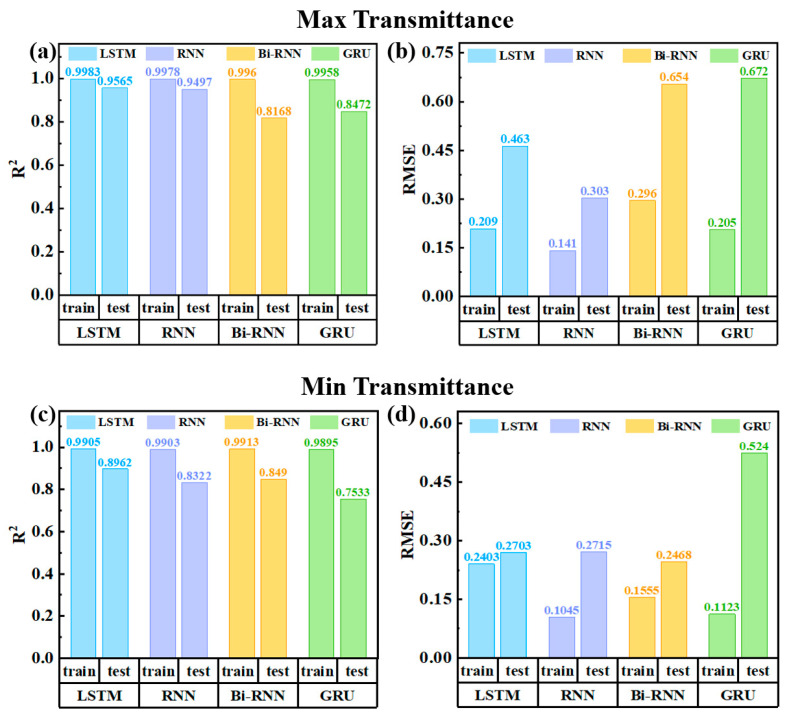
Comparison of prediction results for LSTM, RNN, Bi-RNN, and GRU Models: (**a**) R^2^ of max transmittance, (**b**) RMSE of max transmittance, (**c**) R^2^ of min transmittance, and (**d**) RMSE of min transmittance.

Figure 5 presents the average performance metrics of the model training and test sets for the four methods. The specific R^2^ and RMSE regarding each model at different D_2_O ratios are provided in Tables S2–S4 of the ESI. LSTM excels in forecasting electrochromic device cycle life, effectively capturing transmittance trends. RNN and Bi-RNN are less stable and accurate in testing than LSTM. GRU’s performance is the weakest, needing improvement. LSTM is the top pick for cycle life predictions.

The comparison results show that the LSTM neural network is superior for predicting the cycle life of electrochromic devices, leading to its selection for forecasting. In our experiments, six devices with varying D_2_O concentrations were tested using LSTM models, optimized through a grid search for hyperparameters. From the loss function for the training set curves shown in Figure S4 of the ESI, it can be seen that the loss function tends to stabilize, indicating that it has converged, meaning that the model is trained to a reasonable degree.

All models converged after 20 training iterations. Table 1 provides the metrics for fitting the LSTM model to the cycle life data. The high R^2^ and low RMSE values demonstrate the predictive accuracy and reliability of the model.

**Figure 6:**
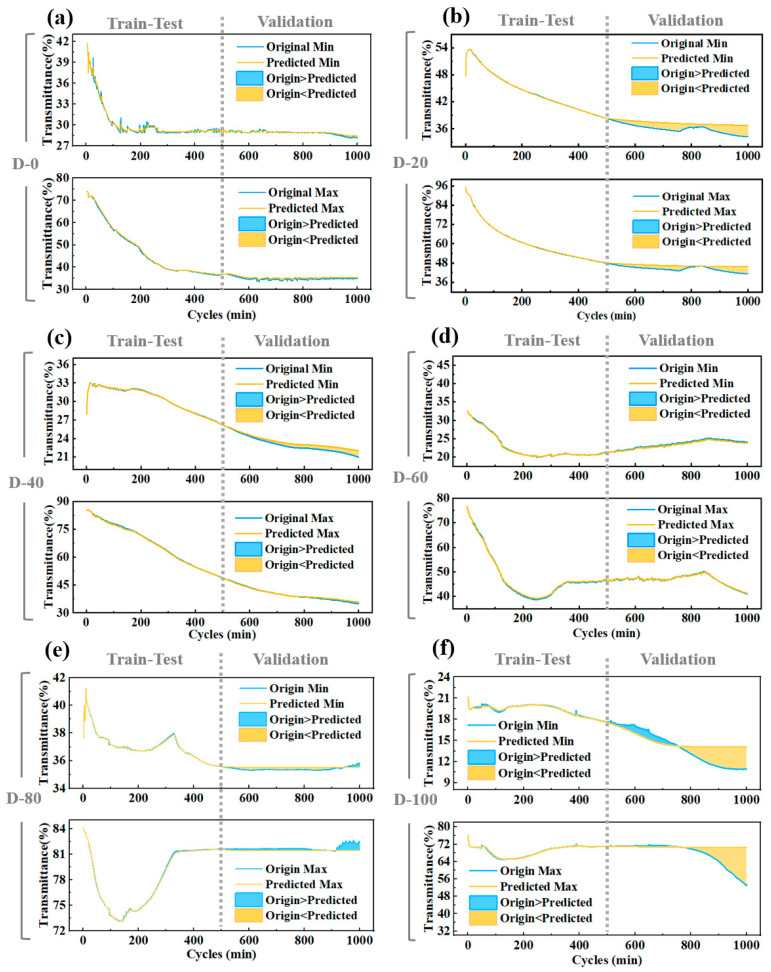
Cycle life prediction results for 6 models (dataset divided according to 500:500). They were trained on datasets collected from (**a**) D-0, (**b**) D-20, (**c**) D-40, (**d**) D-60, (**e**) D-80, and (**f**) D-100.

Figure 6 shows that the LSTM model fits all six electrochromic device concentrations well. However, the prediction accuracy of some concentration groups was lower than expected, especially after more than 700 cycles. This can be partly attributed to uncertainty in experimental operation, systematic errors, differences in device preparation and testing environments, differences in long-term device stability, and environmental fluctuations during experiments.

Additionally, to ascertain whether this observation is associated with the division ratio of the dataset, we proceeded to subdivide the training-test set and validation set in accordance with the ratio of 750:250 and reconstructed the model. The results (see Figure S5 and Table S5) demonstrate that the prediction bias persists even with the revised division of the dataset. This indicates that the dataset’s division method is not the main cause of the fitting discrepancy.

In order to deeply analyze the causes of prediction bias, we introduce the LIME method. LIME is a versatile approach for analyzing the interpretability of machine learning models. This method involves training an interpretable machine learning model using understandable features and then fitting a local linear model in the neighborhood of a specific sample to obtain feature weights of the original model. By analyzing these feature weights, LIME quantifies feature importance and assesses the impact of individual features on the model’s predictions for specific samples.

**Figure 7:**
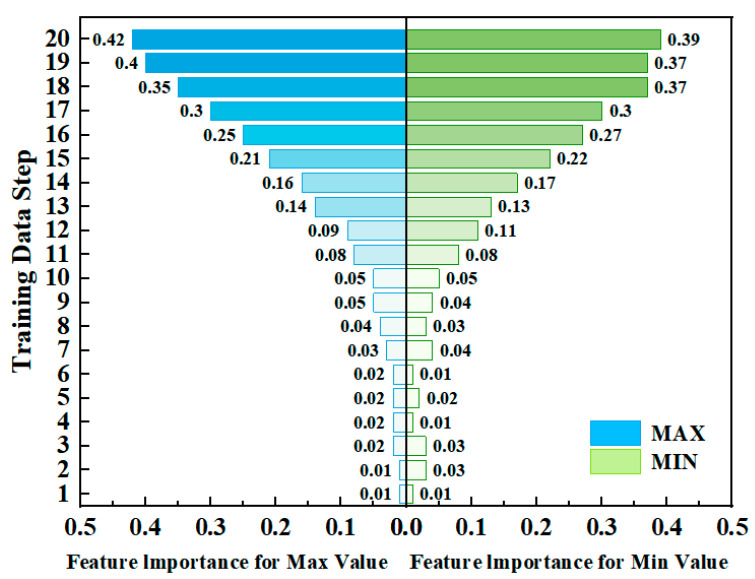
LIME analysis of LSTM: feature importance for predicting output values with D-40.

We applied the LIME method to analyze which factors influence the output values of a LSTM neural network. As shown in Figure 7, in the LSTM neural network with a 40% heavy water doping ratio, both the maximum and minimum values are primarily influenced by the 13th to 20th groups of training data within the sliding window. The importance weights for the 20th input group reach as high as 0.42 and 0.39, respectively, indicating that the model effectively captures the transmittance variation trends caused by chemical changes in electrochromic devices during cyclic testing. It also selectively disregards the influence of older historical data.

These findings further emphasize that statistical metrics such as the coefficient of R^2^ and RMSE should not be solely relied upon in modeling cycle life prediction of electrochromic devices but should be comprehensively evaluated in conjunction with interpretable analyses of feature significance.

In conjunction with the results of model construction, we performed an in-depth analysis of the time periods with significant prediction deviations. For D-0, the model showed poor performance due to H_2_O causing strong hydrolysis, memory effects, and charge buildup, leading to only a general downward trend being predictable and small fluctuations being hard to forecast. Instability due to hydrolysis was also observed in D-20 and D-40. For devices with a high percentage of D_2_O, the late changes are related to the difficulty of electrolyzing D_2_O and the nature of its electrolytic equilibrium. The properties of D_2_O allow the devices to withstand higher voltages. Still, at the same time, the devices are more susceptible to damage at high voltages, resulting in abnormal performance degradation. These analyses are consistent with our previous analyses of D_2_O mixing methods to improve device cycling performance.

The LSTM model is valid despite the discrepancies in the predictions. It was able to capture the characteristics of the cyclic life change of electrochromic devices after long test cycles. This was confirmed by the comparative results of the dataset segmentation and interpretability analysis. This shows that the LSTM model is suitable for predicting the cycle life of electrochromic devices.

### 3.3. Prediction and Validation

We successfully built an LSTM model to predict cycle life for the preliminary experiment data. Due to the regularized concentrations, the model may have limited applicability at unknown ratios. Therefore, we created two validation groups with devices comprising 70% (D-70) and 83% (D-83) of the 10% range before and after 80%.

**Figure 8:**
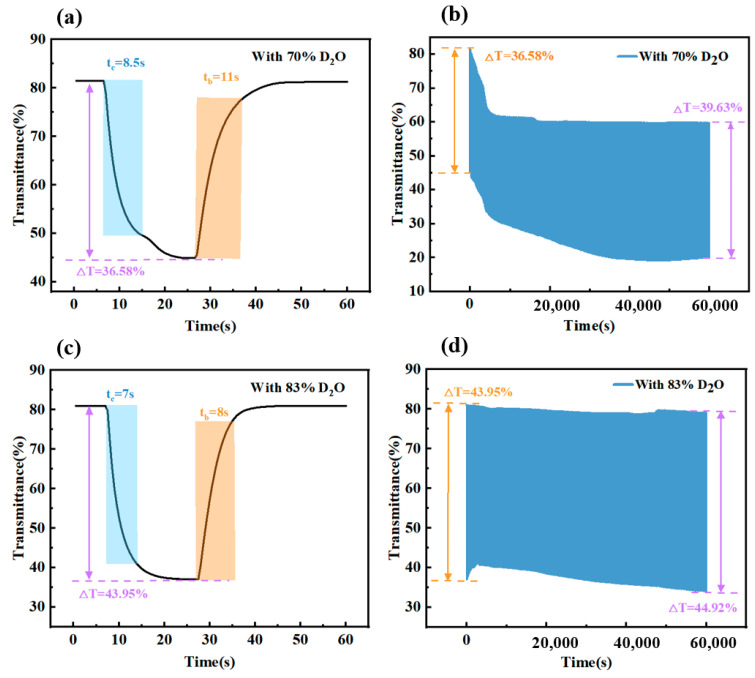
Δ*T*, *t_b_*, and *t_c_* at 700 nm for (**a**) D-70 and (**c**) D-83. Transmittance-time graph for 1000 cycles test for (**b**) D-70 and (**d**) D-83.

As shown in Figure 8, both D-70 and D-83 devices exhibited excellent performance. The D-83 performed better with an initial Δ*T* of 43.95%, a fast response time (*t_c_* = 7 s and *t_b_* = 8 s), and a Δ*T* of 44.92% after 1000 cycles at a current density of 45 A/m². Previous research has examined the relationship between D_2_O mixture concentration and device performance. The cyclic stability of the D-70 and D-83 devices is consistent with these findings. This stability is mainly due to the enhanced hydrogen bonding of D_2_O, which increases the device’s withstand voltage and reduces the hydrolysis reaction. Consequently, the device maintains performance and transitions gradually to a steady state. Table 2 shows some data from similar cycle life studies for comparison. Compared with other devices, our prepared device has the advantage of easy fabrication and simple structure. It balances several fundamental properties, including long cycle life, high modulation capability, fast response, and long-term stability.

**Figure 9:**
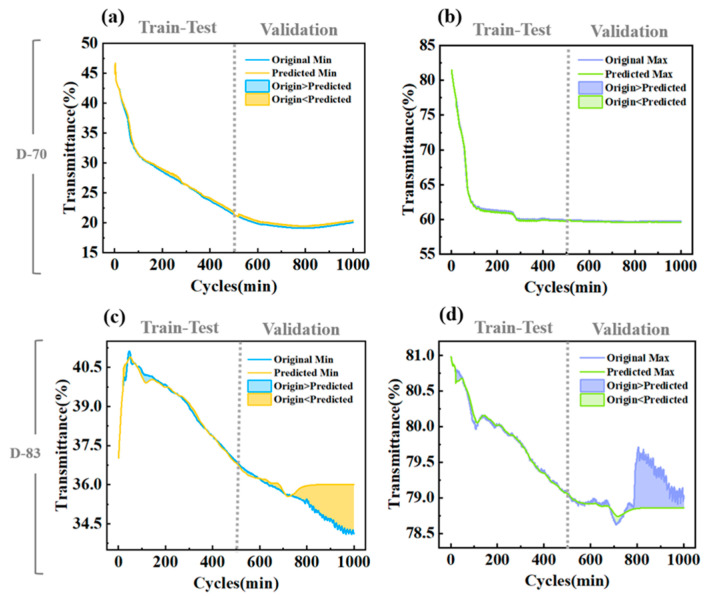
(**a**) Prediction results for the maximum transmittance for (**a**) D-70 and (**c**) D-83. Prediction results for the minimum transmittance for (**b**) D-70 and (**d**) D-83.

To further validate the suitability and accuracy of the model, we used the LSTM model constructed in the previous section for the data of D-70 and D-83. The prediction results are shown in Figure 9, and the R^2^ and RMSE scores are shown in Table 3. The prediction of D-70 almost overlaps with the fitted curve of the original data, indicating a good prediction. In contrast, the maximum deviation of D-83’s model from the actual value occurs after 800 cycles. These fluctuations are challenging for the model to predict and impact prediction accuracy. However, the model demonstrates exceptional performance in predicting the maximum transmittance with high precision and minimal error. These results demonstrate that the LSTM model can be effectively utilized for the prediction of device cycle life, accurately capturing the general trend of cycling performance. With this ML-assisted approach, it is possible to reduce the time cost and expand upon existing experiments significantly.

## 4. Conclusions

In this study, we have employed LSTM modeling and combined it with the interpretable analytical method to predict and analyze the cycle life of devices with different concentration ratios. D-83, prepared in the validation experiments, exhibited excellent performance, with 43.95% initial Δ*T*, a fast response time (*t_c_* = 7 s and *t_b_* = 8 s), and high cyclic stability (Δ*T* = 44.92% after 1000 cycles). The high performance confirms the feasibility of mixing H_2_O and D_2_O as solvents, which can provide a new idea for subsequent inorganic all-liquid devices. The established LSTM model is able to predict future cyclic changes accurately. Therefore, this approach improves efficiency and provides a reliable tool for long-term performance prediction. Our combined device–algorithm approach can provide a reference for future research on the cycle life of electrochromic devices.

## Figures and Tables

**Table 1:** R² and RMSE values for 6 LSTM models (dataset divided according to 500:500).

Devices	Max Transmittance				Min Transmittance			
	R^2^		RMSE		R^2^		RMSE	
	Train-Test	Validation	Train-Test	Validation	Train-Test	Validation	Train-Test	Validation
D-0	0.999	0.976	0.324	0.557	0.982	0.854	0.245	0.104
D-20	0.999	0.976	0.112	0.208	0.999	0.986	0.044	0.104
D-40	0.999	0.988	0.279	0.348	0.999	0.802	0.044	0.536
D-60	0.998	0.903	0.315	0.657	0.998	0.865	0.953	0.106
D-80	0.999	0.928	0.087	0.076	0.998	0.968	0.019	0.032
D-100	0.996	0.968	0.134	0.93	0.967	0.902	0.137	0.740

**Table 2:** Comparison of electrochromic performance between this work and other reported works.

EC Devices	Δ*T* (%) @ Wavelength (nm)	Respond Time *t_c_*/*t_b_* (s)	Cycle	Δ*T* (%) after Cycles	Ref. No
Ammonium metatungstate-iron(II) chloride (With 83% D_2_O)	43.95@700	7.0/8.0	1000	44.92	This work
Zn-W/TiO_2_	66@550	9.0/2.7	1000	60.4	[45]
PB/Li_4_Ti_5_O_12_	55.3@529	40.0/49.2	1000	52.98	[46]
Au/TiO_2_	40@700	6.1/8.1	1000	>35	[47]
Zn/SVO	21@632.8	12.6/25.4	1000	13.02	[48]
polyimide	62@542	6.5/5.1	500	41	[49]
PANI/MXene	55@700	1.3/2.0	500	48	[50]
P-HPA	48@680	2.8/2.4	300	40.6	[51]
MXene/WO_3-x_	60.4@660	12.0/8.0	200	48.8	[52]
WO_3_/ZIF-67 MOFs	30.1@650	33.0/30.0	100	>25	[53]

**Table 3:** R² and RMSE values of the LSTM model for D-70 and D-83.

Devices	Max Transmittance				Min Transmittance			
	R^2^		RMSE		R^2^		RMSE	
	Train-Test	Validation	Train-Test	Validation	Train-Test	Validation	Train-Test	Validation
D-70	0.991	0.980	0.335	0.656	0.835	0.608	0.115	0.297
D-83	0.988	0.999	0.047	0.026	0.584	0.674	0.178	0.426

## Data Availability

The data presented in this study are available on request from the corresponding author.

## Supplementary Materials

The following supporting information can be downloaded at: https://www.mdpi.com/article/10.3390/mi15091073/s1, Figure S1: (a) The confocal laser scanning microscope (CLSM) image of the device sample. (b) The optical transmittance spectra of the device in the initial, colored, and bleached state; Figure S2: Δ*T* change graphs during 1000 cycle life tests for 6 devices: (a) D-0, (b) D-20, (c) D-40, (d) D-60, (e) D-80, (f) D-100; Figure S3: Schematic diagram of device reaction principle; Figure S4: Loss function curves for the training set; Figure S5: Cycle life prediction results for 6 models (dataset divided according to 750:250). They were trained on datasets collected from (a) D-0, (b) D-20, (c) D-40, (d) D-60, (e) D-80, and (f) D-100; Table S1: Transmittance data before and after stability; Table S2: Values of R^2^ and RMSE in RNN models; Table S3: Values of R^2^ and RMSE in GRU models; Table S4: Values of R^2^ and RMSE in Bi-RNN models; Table S5: R^2^ and RMSE values for 6 LSTM models (dataset divided according to 750:250).
