# Supplementary material for: Machine Learning-Guided Cycle Life Prediction for Electrochromic Devices Based on Deuterium and Water Mixing Solvent
Source: Micromachines (Basel). 2024 Aug 26;15(9):1073. doi: 10.3390/mi15091073 (PMC11433885; doi:10.3390/mi15091073)
Supplement: Supplementary file 1 [file micromachines-15-01073-s001.zip › micromachines-3160990-supplementary.pdf]

micromachines

MDPI

Supplementary Materials

Machine Learning-Guided Cycle Life Prediction for Electrochromic Devices Based on Deuterium and Water Mixing Solvent

Yitong Wu [1,2,†], Sifan Kong [3,†], Qingxin Yao [1], Muyun Li [4,*], Huayi Lai [5], Duoyu Sun [3], Qingyue Cai [4], Zelin Qiu [2], Honglong Ning [4], and Yong Zhang [2,*]

[1] School of Electronics and Information Engineering, South China Normal University, Foshan 528225, China; yitongwu@m.scnu.edu.cn (W.Y.); 20213903020@m.scnu.edu.cn (Y.Q.)

[2] School of Semiconductor Science and Technology, South China Normal University, Foshan 528225, China; 20225031032@m.scnu.edu.cn (Q.Z.)

[3] School of Software, South China Normal University, Foshan 528225, China; kongsifanscnu@foxmail.com (K.S.); 20222034018@m.scnu.edu.cn (S.D.)

[4] State Key Laboratory of Luminescent Materials and Devices, School of Materials Science and Engineering, South China University of Technology, Guangzhou 510640, China; 13922705035@163.com (C.Q.); ninghl@scut.edu.cn (N.H.)

[5] Aberdeen Institute of Data Science and Artificial Intelligence, South China Normal University, Foshan 528225, China; 20213802071@m.scnu.edu.cn (L.H.)

* Correspondence: llmyscut@163.com (L.M.); zycq@scnu.edu.cn (Z.Y.)

† These authors contributed equally to this work.

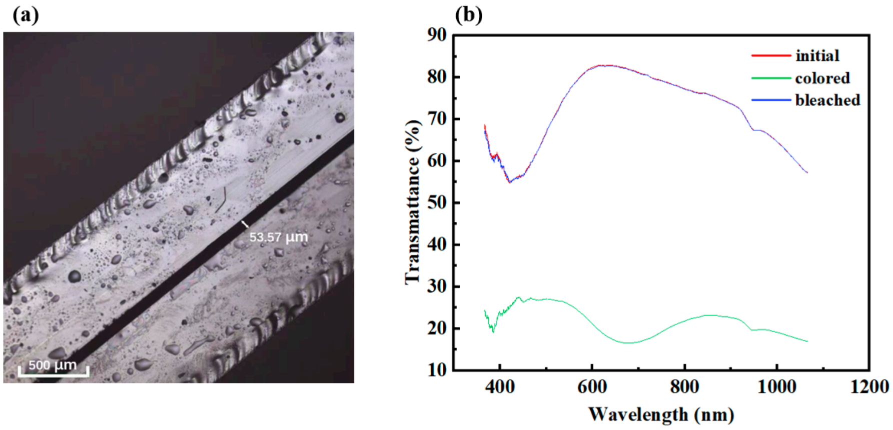

Figure S1. (a) The confocal laser scanning microscope (CLSM) image of the device sample. (b) The optical transmittance spectra of the device in the initial, colored, and bleached state.

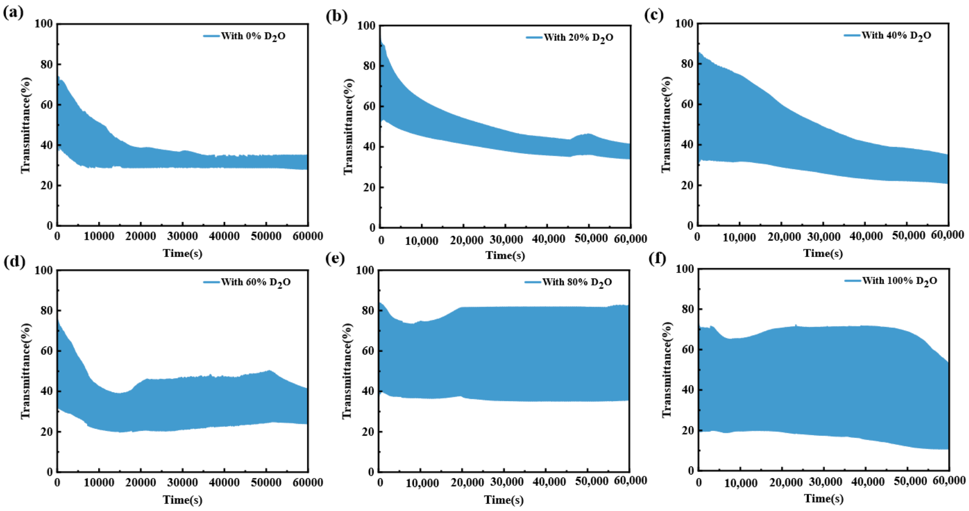

Figure S2. ΔT change graphs during 1000 cycle life tests for 6 devices: (a) D-0, (b) D-20, (c) D-40, (d) D-60, (e) D-80, and (f) D-100.

Table S1. Transmittance data before and after stability.

EC devices	Initial ΔT (%)	ΔT after 1000 cycles (%)	ΔT retention (%)
D-0	32.36	6.88	21.26
D-20	47.52	7.02	14.77
D-40	57.4	14.01	24.41
D-60	44.23	16.99	38.41
D-80	46.42	46.64	100.47
D-100	54.78	42.06	76.78

Figure S2 illustrates the transmittance changes of the six devices with varying D_2O to H_2O mixing ratios (0% to 100%) during 1000 cycle life tests. Despite the disparate mixing ratios, all devices exhibit comparable performance degradation trends in the initial stages, particularly around 20000 s, which represents one-third of the total number of cycles. The ΔT of D-0, D-20, and D-40 exhibited a gradual decrease and did not recover. In contrast, D-60, D-80, and D-100 all rebounded and stabilized after a period of decline, with D-80 being the most stable, exhibiting the least degree of change in the transmittance stabilization in both the colored and bleached states. These differences can be attributed to the effective inhibition of the hydrogen precipitation reaction by D_2O, a conclusion consistent with Li et al.'s findings [14].

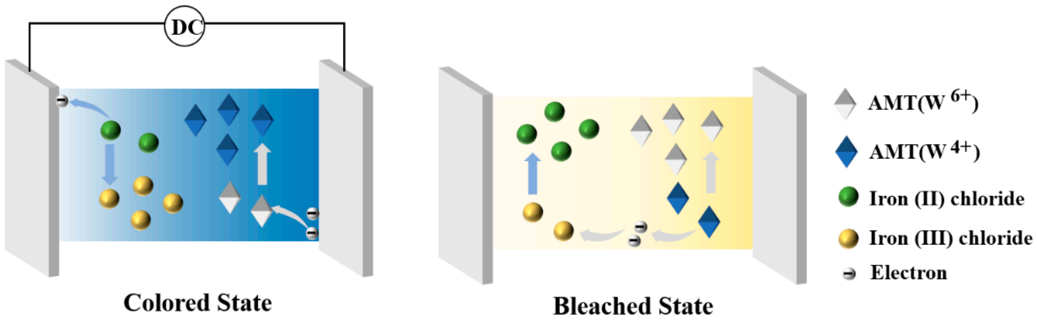

Figure S3. Schematic diagram of device reaction principle.

Figure S3 illustrates the color change mechanism of the electrochromic device. The cyclic reaction process of electrochromism involves the coloring of the device when the metatungstate ion undergoes an ionic reduction to blue, and the ferrous ion is oxidized to become a ferric ion. The coloring process is enabled to proceed rapidly by the combined effect of external voltage and Fe^{2+} ions, while the fading process proceeds spontaneously without external excitation, resulting in a more rapid coloring phase than the fading phase. Upon the removal of the external electric field, the ions diffused due to the concentration gradient, and the trivalent iron ions combined with the metatungstate ions in the reduced state undergo a redox reaction, which resulted in the fading of the metatungstate ions. Since the device is in a liquid state, a hydrogen precipitation reaction will inevitably occur when a voltage is applied. Consequently, the electrochromic reaction is accompanied by the electrolysis of H_2O and D_2O, which affects the behavior of the metatungstate ion [14, 30, 42].

Table S2. Values of R^2 and RMSE in RNN models.

Devices	Max Transmittance				Min Transmittance			
	R^2		RMSE		R^2		RMSE	
	Train-Test	Validation	Train-Test	Validation	Train-Test	Validation	Train-Test	Validation
D-0	0.999	0.814	0.16	0.238	0.977	0.682	0.238	0.152
D-20	0.999	0.969	0.079	0.255	0.999	0.947	0.046	0.203
D-40	0.999	0.981	0.235	0.449	0.996	0.535	0.117	0.822
D-60	0.999	0.98	0.113	0.291	0.999	0.985	0.069	0.125
D-80	0.999	0.967	0.062	0.051	0.997	0.859	0.036	0.04
D-100	0.992	0.987	0.197	0.535	0.974	0.985	0.121	0.287

Table S3. Values of R^2 and RMSE in GRU models.

Devices	Max Transmittance				Min Transmittance			
	R^2		RMSE		R^2		RMSE	
	Train-Test	Validation	Train-Test	Validation	Train-Test	Validation	Train-Test	Validation
D-0	0.999	0.84	0.164	0.22	0.984	0.907	0.232	0.082
D-20	0.999	0.914	0.175	0.43	0.999	0.95	0.046	0.197
D-40	0.999	0.98	0.167	0.46	0.988	0.76	0.073	0.45
D-60	0.998	0.816	0.309	0.886	0.998	0.617	0.132	0.643
D-80	0.998	0.66	0.112	0.167	0.991	0.819	0.071	0.046
D-100	0.982	0.873	0.304	1.87	0.977	0.467	0.12	1.726

Table S4. Values of R^2 and RMSE in Bi-RNN models.

Devices	Max Transmittance				Min Transmittance			
	R^2		RMSE		R^2		RMSE	
	Train-Test	Validation	Train-Test	Validation	Train-Test	Validation	Train-Test	Validation
D-0	0.999	0.913	0.077	0.162	0.98	0.884	0.26	0.092
D-20	0.996	0.309	0.564	1.222	0.994	0.862	0.297	0.329
D-40	0.997	0.744	0.553	1.679	0.994	0.399	0.147	0.773
D-60	0.999	0.987	0.215	0.23	0.999	0.989	0.094	0.105
D-80	0.998	0.96	0.109	0.057	0.997	0.965	0.041	0.02
D-100	0.987	0.988	0.255	0.575	0.984	0.995	0.094	0.162

As can be seen from the scores shown in Table S2-S4, the LSTM model performs best in terms of R^2, particularly when predicting the maximum transmittance. However, in

terms of RMSE, the LSTM model is not the lowest on the test set. For instance, when predicting the minimum transmittance, the RMSE of the Bi-RNN neural network model on the test set (0.2468) is lower than the RMSE of the LSTM model (0.2703). This indicates that although the LSTM model demonstrates superior performance in most cases, other models, such as the Bi-RNN, may also exhibit comparable or even superior performance in specific performance metrics.

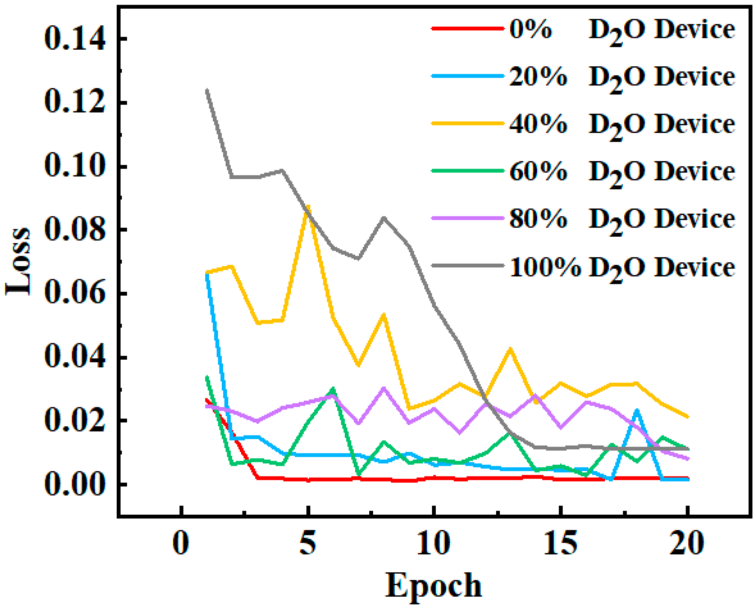

Figure S4. Loss function curves for the training set.

Table S5. R^2 and RMSE values for 6 LSTM models (dataset divided according to 750:250).

Devices	Max Transmittance				Min Transmittance			
	R^2		RMSE		R^2		RMSE	
	Train-Test	Validation	Train-Test	Validation	Train-Test	Validation	Train-Test	Validation
D-0	0.999	0.984	0.146	0.1968	0.405	0.821	0.153	0.121
D-20	0.999	0.999	0.133	0.033	0.912	0.972	0.488	0.128
D-40	0.999	0.999	0.127	0.085	0.796	0.898	0.517	0.136
D-60	0.999	0.999	0.163	0.075	0.995	0.879	0.187	0.118
D-80	0.999	0.999	0.078	0.024	0.883	0.963	0.135	0.026
D-100	0.996	0.998	0.134	0.071	0.889	0.102	1.832	0.846

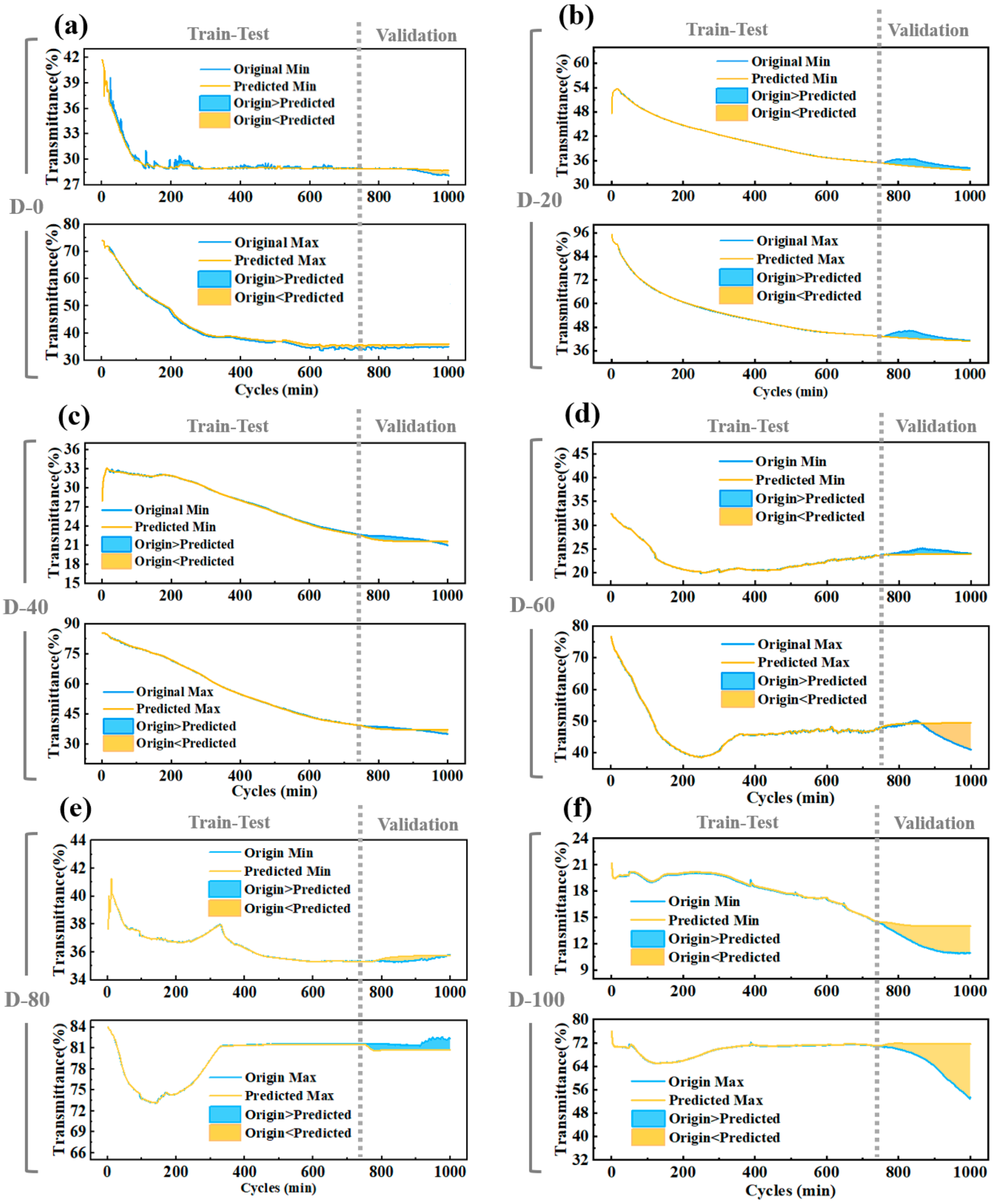

Figure S5. Cycle life prediction results for 6 models (dataset divided according to 750:250). They were trained on datasets collected from (a) D-0, (b) D-20, (c) D-40, (d) D-60, (e) D-80, and (f) D-100.
